# O’nyong-nyong Virus Infection Imported to Europe from Kenya by a Traveler

**DOI:** 10.3201/eid2010.140823

**Published:** 2014-10

**Authors:** Dennis Tappe, Annette Kapaun, Petra Emmerich, Renata de Mendonca Campos, Daniel Cadar, Stephan Günther, Jonas Schmidt-Chanasit

**Affiliations:** World Health Organization Collaborating Centre for Arbovirus and Haemorrhagic Fever Reference and Research, Hamburg, Germany (D. Tappe, P. Emmerich, D. Cadar, S. Günther, J.Schmidt-Chanasit);; University Medical Center, Heidelberg, Germany (A. Kapaun);; Federal University of Rio de Janeiro, Rio de Janeiro, Brazil (R. de Mendonca Campos);; German Centre for Infection Research, Partner Site Hamburg-Luebeck-Borstel, Hamburg (S. Günther, J. Schmidt-Chanasit)

**Keywords:** O’nyong-nyong, viruses, Kenya, Germany, mosquito, PCR, Chikungunya

**To the Editor:** O’nyong-nyong virus (ONNV) is a mosquitoborne RNA virus of the *Togaviridae* family. The virus was first isolated in June 1959 from serum samples from febrile patients in the northern province of Uganda (*1*). Unlike ONNV is primarily transmitted by anopheline mosquitoes (*2*). ONNV is genetically and serologically related to chikungunya virus (CHIKV) (*1*), but is restricted to the African continent. The clinical picture resembles CHIKV infection, i.e., a self-limited febrile illness characterized by headache, rash, and joint pain. In contrast to CHIKV, ONNV is reported to cause lymphadenopathy more often and affected joints do not show effusions (*3*).

ONNV caused 2 large-scale epidemics in East Africa during 1959–1962 and in 1996. The first instance had spread from Uganda south to Mozambique and westward to Senegal. Comprising >2 million cases in east Africa alone, this first epidemic ranked among the largest mosquitoborne virus outbreaks recorded (*4*). After an absence of reported cases for 35 years, a second ONNV epidemic occurred in Uganda (*3**–**4*). Patients had fever, a maculopapular rash, pruritis, myalgia, and arthralgia of large joints. Lymphadenitis, most often of the posterior cervical spine region, was also observed (*3*). Despite the virus’ potential to cause large outbreaks and its endemicity in the vast geographic area of East Africa, and at least sporadic occurrence in West Africa, imported cases to other areas have not been reported.

On October 14, 2013, a 60-year-old woman residing in Germany who had returned home 2 days before from a 7-week vacation in East Africa sought medical attention at the University Medical Center, Section of Clinical Tropical Medicine, in Heidelberg for recurring fever and illness that began during her travel. She and her husband had traveled from Kenya to Uganda, Rwanda, Tanzania, and back to Kenya, along the shore of Lake Victoria. Bed nets and malaria prophylaxis were used regularly. On October 9, she had experienced the first episode of fever, general malaise, arthralgia, and nausea while staying at the lake shore near the city of Kisumu, Kenya. Fever had persisted until October 12. Thin and thick blood films, examined in a local hospital and later in Nairobi, did not show malarial parasites.

October 14 was day 5 of symptom onset. Her fever reached 39°C and lasted 3 more days. It was accompanied by cervical spine and nuchal lymphadenopathy, nausea, and arthralgia of the small joints of her hands and feet. A maculopapular rash developed, which covered her face, hands, feet, and trunk. Her face, hands, and feet were edematous. Laboratory tests on admission to the medical center revealed a slightly elevated C-reactive protein level of 13 mg/L (reference level<5). Full blood count and results of liver function tests were within reference ranges. Thin and thick blood films were examined again and were negative for *Plasmodium* spp. A serum sample from the day of admission showed anti-ONNV IgM and IgG and anti-CHIKV IgM and IgG in the indirect immunofluorescence assay, according to Tappe et al. (*5*, Table). Serology for dengue virus and generic alphavirus reverse transcription PCR (*6*) were negative. A 4-fold anti-ONNV IgG titer decrease in the indirect immunofluorescence assay was demonstrated in the second serum sample, which was collected 26 days after disease onset (Table). The presence of ONNV-specific neutralizing antibodies in the second serum sample was confirmed by a virus neutralization test. Cross-neutralizing antibodies against CHIKV were detected also, but with a notably lower titer (1:80) when compared with the ONNV titer (1:1,280) (Table). Ten days after symptom onset, the patient recovered spontaneously. Her husband had no symptoms of illness during travel or after returning.

**Table:** Results of serologic analysis of a German traveler from Kenya with O’nyong-nyong virus infection, October 2013*

Virus	Immunofluorescence assay†						Virus neutralization test, 26 d after symptom onset
	5 d after symptom onset			26 d after symptom onset			
	IgG	IgM		IgG	IgM		
O’nyong-nyong	1:160	1:160		1:2,560	1:1,280		1:1,280
Chikungunya	1:80	1:80		1:2,560	1:320		1:80
Sindbis	Neg	Neg		1:640	Neg		ND
Semliki Forest	Neg	Neg		Neg	Neg		ND


*Neg, negative; ND, not done. †Immunofluorescence assay and virus neutralization test results of <1:20 were considered negative.

We report the laboratory-confirmed case of an ONNV infection imported into Europe. This patient most likely was infected in the eastern part of Kenya (Kisumu region), where she had stayed during the 2 weeks before symptom onset. The case highlights the fact that ONNV infections, which occur sympatrically with CHIKV infections in East Africa, lead to symptoms resembling CHIKV infection. The clinical and laboratory findings emphasize the importance of a careful diagnostic and clinical assessment of travelers with suspected arboviral disease, and consideration of the well-known serologic cross-reactions in the alphavirus group. Because of the serologic and clinical similarities of ONNV and CHIKV infections, it remains unclear how many true ONNV infections in travelers have been diagnosed as CHIKV infections. Similar to other arboviruses, especially CHIKV and dengue viruses (*7**,**8*), ONNV might have the potential to spread to areas outside of Africa. There are no known invasive anopheline vectors for ONNV in Europe, but it was demonstrated that the culicine mosquito species *Aedes aegypti*, found in some parts of Europe (*8*), might be a competent vector for ONNV (*9*). Thus, it will be critical to study the vector competence of both the indigenous anopheline and culicine mosquitoes for ONNV in Europe.
